# Pregnancy Complications and Feto-Maternal Monitoring in Rabbits

**DOI:** 10.3390/vetsci10100622

**Published:** 2023-10-17

**Authors:** Patrícia Pinto-Pinho, Maria de Lurdes Pinto, José Monteiro, Margarida Fardilha, Rosário Pinto-Leite, Bruno Colaço

**Affiliations:** 1 Centre for the Research and Technology of Agro-Environmental and Biological Sciences, University of Trás-os-Montes and Alto Douro, 5000-801 Vila Real, Portugal; bcolaco@utad.pt; 2 Laboratory of Signal Transduction, Institute of Biomedicine, Department of Medical Sciences, University of Aveiro, 3810-193 Aveiro, Portugal; mfardilha@ua.pt; 3 Laboratory of Genetics and Andrology, Centro Hospitalar de Trás-os-Montes e Alto Douro, E.P.E, 5000-508 Vila Real, Portugal; mlleite@chtmad.min-saude.pt; 4 Animal and Veterinary Research Centre, University of Trás-os-Montes and Alto Douro, 5001-801 Vila Real, Portugal; lpinto@utad.pt; 5 José Azevedo Monteiro, Lda., 4625-679 Vila Boa do Bispo, Portugal; geral.zmvet@gmail.com

**Keywords:** *Oryctolagus cuniculus*, Maternal diseases, Gestation, Development abnormalities, Fetal monitoring, Ultrasonography

## Abstract

**Simple Summary:**

Rabbit farming plays a significant role in the global production of animal-derived protein, with hundreds of millions of rabbits being produced every year. However, research on feto-maternal monitoring in rabbits remains limited. This review aims to explore intricate factors affecting maternal and fetal health, providing valuable insights for farmers and researchers alike. Standardizing monitoring approaches, such as ultrasonography and other screening methods, and establishing reference parameters would enable more effective tracking of fetal well-being. This, in turn, would facilitate differentiation between healthy and unhealthy rabbits, offer guidance for treatments, and enhance rabbit welfare and reproductive efficiency, thereby holding substantial promise for the rabbit farming industry.

**Abstract:**

Rabbit production holds significant relevance in modern agriculture due to its potential as a sustainable source of high-quality protein and efficient feed conversion, contributing to food security and economic diversification. Nevertheless, studies incorporating feto-maternal monitoring in this species are uncommon. This review gathers research on the monitoring and evaluation of factors affecting rabbit gestation, providing a better understanding of the causes of prenatal development abnormalities. These include studies regarding how chronic maternal hypertension, gestational diabetes, maternal stress, ectopic gestation, maternal uterine ischemia and fetal hypoxia, intrauterine growth restriction, superfetation, maternal age, maternal nutritional status, maternal physical condition, maternal and embryonic genotype, and the intrauterine location of rabbit fetuses can potentially impact rabbits’ reproduction and maternal and fetal health. Among other monitoring techniques, ultrasonography, considered one of the best tools for diagnosing pregnancy and conducting follow-up, is also reviewed. Details on measurable fetal-development parameters in rabbits and precautions to be considered before and during the examination are also provided. Additional studies are required to understand why some events occur and their consequences throughout gestation, allowing the determination of new biomarkers or cut-offs that can be helpful for early diagnosis and improve reproductive efficiency.

## 1. Introduction

Rabbits remain a solid, cost-effective alternative for meat producers compared to more traditional sources of animal protein such as pork and beef, not only due to the nutritional composition of their meat and lower environmental impact but also because rabbits have great reproductive characteristics [1,2]. They have a rapid growth rate, short puberty and gestation cycles, large litters, and are continuous breeders [2]. According to the Food and Agriculture Organization, between 2010 and 2020, a mean of 1,156,840 ± 182,290 tonnes of rabbit meat was produced, and 793,863,000 ± 107,399,917 animals were slaughtered for meat consumption each year [3]. Several campaigns and recent studies aimed at understanding consumers’ attitudes toward rabbit meat could also contribute to market development in the following years [4,5].

In the realm of rabbit farming (cuniculture) and reproductive techniques, proper management during pregnancy remains crucial to ensure the health of both female rabbits (does) and their offspring (kits). To this end, understanding the complex dynamics of embryonic development and maternal interactions plays a pivotal role in improving reproductive efficiency.

Rabbits stand out among domestic species due to their hemochorial placentation, which strongly resembles the human placental structure, and because their maternal spiral arteries undergo a transformation similar to that which occurs in humans [6,7]. In addition to the possibility of performing artificial insemination, it is worth noting that successive blood sampling can be conducted and that a doe’s uterus is duplex, allowing for the introduction of embryos with diverse genetic backgrounds into a single doe simultaneously. This facilitates the evaluation of how interactions between fetal and maternal factors can impact prenatal and postnatal outcomes (reviewed in [8]).

Conducting follow-up studies holds great potential for reducing the risk of fetal mortality and enhancing our understanding of fetal growth disorders and their underlying causes [9]. However, while gestational monitoring is common in other domestic species, research on gestational monitoring in rabbits is limited, with most studies focused on using rabbits as animal models [10,11,12,13].

In addition to reproductive problems that are commonly attributed to infectious diseases, rabbit reproduction and fetal development can be influenced by various other maternal chronic and acute conditions and factors that encompass the nutritional status of the doe and handling and housing conditions [14,15,16,17,18,19,20,21,22]. Thus, it is important for producers to be aware of these potential issues and explore possible solutions for early diagnosis and/or treatment to ensure the well-being and reproductive success of their rabbits.

Therefore, this review aims to address some medical conditions described in does during the gestation period and their implications on rabbits’ fetal development and health. Additionally, some feto-maternal monitoring techniques will be approached, and the applications of one of the most complete techniques, ultrasonography, will be further explored in rabbits.

## 2. Medical Conditions during Pregnancy: How Do They Affect Its Course?

After the successful implantation of a fertilized egg, the doe will host a complex series of events. For 31 days, on average, about 4–5 or 8–12 fetuses will develop in smaller or larger breeds, respectively [18]. Placental and fetal development in rabbits was already reviewed by Lopez-Tello [8]. However, this process does not depend exclusively on successful copulation or insemination. Pregnant does can suffer from a series of acute or chronic conditions that compromise fetal development. Important health conditions regarding their implications for pregnancy are described below and summarized in Supplementary Table S1.

### 2.1. Chronic Maternal Hypertension

In a previous study, it was reported that normal rabbits typically exhibit blood pressure within the range of 70 to 170 mm Hg. Concurrently, the pulse rate, measured simultaneously with blood pressure, demonstrated variability, spanning from 112 to 300 beats per minute [23]. Maternal hypertension in rabbits has already been described, particularly in studies that used them as animal models [8]. To induce mild or moderate hypertension, one (2K-1W) or two (2K-2W) kidneys are commonly exposed and wrapped in cellophane, respectively. According to McArdle and collaborators [24], hypertension disturbs the differentiation and gene expression of the placenta, namely placental 11β-hydroxysteroid dehydrogenase type 2 mRNA and components of the renin-angiotensin system that play an important role in the development of the fetal cardiovascular system (angiotensin receptors type 1 and 2 mRNA).

Even though hypertension leads to a decrease in the proportion of the placenta occupied by the trophoblast, vascular alterations seem to counterbalance it, helping to maintain fetal weight [24]. In another study, when compared to normotensive does, mild (+15 mmHg) and moderate (+25 mmHg) hypertensive does presented increased proportion and volume of placental fetal capillaries in early-mid- and late-gestation, respectively, allowing the transfer of nutrients to the fetuses [24]. Another study has also suggested that the transfer of nutrients may be modified according to the severity of hypertension [25]. In late-gestation, when compared to normotensive does, mild hypertensive does had similar placental blood flow (PBF) but increased placental efficiency, while moderate hypertensive does had reduced PBF, with no changes in fetal or placental weight or efficiency observed [25].

Yet, if the placental functional capacity is affected by hypertension, the development of fetuses or their health may be impaired. Certain outcomes may be sex-related. A previous study showed that only female offspring of pregnant does with 2K-1W hypertension had increased blood pressure in adulthood [26]. The hypothalamic–pituitary–adrenocortical axis of prenatal programming may differ between females and males, promoting different responses to the same stimulus [24].

It is also possible to track the growth trajectory of fetuses using ultrasound. A recent study showed that it differed between fetuses born to 2K-1W and 2K-2W hypertensive does and reflected a smaller litter size in the first case. Ultrasound data have also demonstrated that hypertensive fetuses had a higher fetal heart rate, kidney length (26 gestation days), and biparietal diameter than normotensive fetuses [10]. Further studies focused on monitoring gestation would provide a good starting point for a better understanding of the possible consequences of hypertension on offspring growth.

In humans, some other complex medical disorders may arise with hypertension, such as preeclampsia. Preeclampsia is typically linked to a sudden onset of hypertension along with proteinuria during gestation or to gestational hypertension coupled with other symptoms that can evolve into eclampsia (as cited by [7]). While eclampsia is described in small animals, namely dogs, to our knowledge, there have been no reports of the natural occurrence of these disorders in rabbits; it has only been documented as an animal model [7,27]. In a study where pregnancy toxemia was induced by constricting the aorta below the renal arteries, this procedure effectively reduced blood supply significantly below the constriction point. This reduction in blood supply was accompanied by hypertension, proteinuria, increased maternal weight, and a decrease in fetal weight [28].

### 2.2. Gestational Diabetes

The doe is diagnosed with gestational diabetes mellitus when it begins to show elevated glucose levels, namely in cases where insulin resistance is not compensated by sufficient insulin production [19]. Normal values of glucose in rabbits can vary slightly between studies and species. For New Zealand White rabbits, whole-blood and plasma glucose levels range between 3.7 and 10.2 mmol/L, with whole-blood glucose levels generally lower than plasma levels (as reviewed in [29,30]). In Japanese White rabbits, some authors have reported blood glucose values between 5.6 and 7.8 mmol/L [31]. However, it is important to note that factors other than diabetes can also influence blood glucose levels, such as stress [29].

In 1981, the first case of spontaneous diabetes mellitus in rabbits was reported [32]. The authors observed polydipsia and polyuria in a female New Zealand white rabbit when it was 6–12 months old, which subsequently revealed fasting hyperglycemia (540–590 mg/dL) and glycosuria (50 g per 24 h) [32]. The spontaneous diabetic colony produced three distinct groups of offspring: rabbits with normal glucose disposal, rabbits with abnormal glucose disposal without fasting hyperglycemia, and rabbits with abnormal glucose disposal with hyperglycemia. Additionally, the diabetic colony exhibited significantly smaller litter sizes and higher mortality rates up to 12 weeks of age when compared to the norms reported for New Zealand White rabbits. Notably, hyperglycemia could be effectively corrected with insulin treatments [32]. This condition can, therefore, affect embryonic development.

As demonstrated by Thieme and collaborators [33], both insulin and insulin-like-growth-factor 1 are potent growth factors and necessary for mesoderm formation in blastocysts. In their study, the blastocysts of does with diabetes showed a retarded growth, probably related to an impaired expression of Wnt signaling molecules and a mesoderm-specific transcription factor (Brachyury) [33]. More recently, Rousseau-Ralliard et al. [34] induced diabetes type-1 seven days before coitum in does. Four days post-coitum, they transferred the embryos to does with normal glycemia. This minimal exposure until the blastocyst stage was sufficient to induce irreversible structural and molecular adaptations of the placenta and retarded growth, dyslipidemia, and hyperglycemia in fetuses [34].

In rabbits, excess weight has already been associated with a greater predisposition to insulin resistance during pregnancy, probably related to a reduction in insulin sensitivity [19]. Thus, the weight at the onset of gestation and the type of diet during its course are critical to preventing diabetes. Although the underlying mechanisms are not yet well known, supplementation with fish oil, an ingredient rich in long-chain polyunsaturated fatty acids (EPA and DHA), improved insulin sensitivity in pregnant does [19]. Compared to controls, EPA and DHA did not promote changes in body fat, but their anti-inflammatory properties may play a role in reversing adipose inflammation through down-regulation of genes related to immune response pathways, such as tumor necrosis factor-alpha, nuclear factor-kappa B, or toll-like receptors [35]. Among others, it is also possible that they lead to an enhancement of the number of insulin receptors and insulin affinity [36].

### 2.3. Ectopic Gestation

The term “ectopic gestation”, or “extrauterine gestation”, describes an uncommon condition in mammals in which implantation and development of the fertilized egg occur outside the main cavity of the uterus [20,37]. In this situation, gestation can develop in the oviduct, ovary, cervix, or abdominal-peritoneal cavity [37].

Despite the few reports available, ectopic gestation has already been described in wild lagomorphs, farmed, laboratory, and pet rabbits. Nevertheless, few or no reproductive abnormalities were observed [20,37,38,39,40,41].

Most case reports describe an extrauterine abdominal gestation (AG), which can be primary (oocytes accidentally released from the fimbria into the peritoneal cavity) or secondary (oocytes released into the abdomen due to uterine or tubal trauma) [37]. However, ectopic gestation is rarely discovered due to the lack of clinical signs in most cases. According to the reports on this subject, the diagnosis is often based on the presence of palpable abdominal mass(es) and/or further discovery during the necropsy of at least one fetus that can be calcified (lithopedia) or not, still attached to an inflamed uterine horn with fibrosis by a thin fibrovascular stalk or to the abdominal surface by thread-like blood vessels [37,38]. Natural gestation and AG may occur simultaneously [39], as well as new pregnancies can coexist with dead fetuses from previous AG [20,38]. At least two studies have described the identification of fetuses of different gestational ages in pregnant rabbits [20,38]. Abdominal gestation may be somehow linked to impaired mammary gland development, but further studies are needed to understand how [37].

It is worth noticing that this condition appears to be more common in females subjected to artificial insemination. After necropsying 550 fertile females discarded from two Spanish rabbit farms, Segura Gil and collaborators [39] found that about 5% had an AG, mostly secondary. Several hypotheses for this were described by the authors, including the possible influence on tubal motility of pregnant mare serum gonadotropin and gonadotropin-releasing hormone inoculated during artificial insemination (AI) and the possibility of physical injury or perturbation of the reproductive tract induced by deficient manipulation during AI [39]. Many of the mummified fetuses evaluated in these studies were of term size and well developed, suggesting that several of them would be viable if a cesarean section had been performed at the right time [39]. Therefore, systematic monitoring of the pregnant rabbits would be useful to better understand this condition’s etiology and to improve prevention and diagnostic efficiency [38].

### 2.4. Maternal Uterine Ischemia and Fetal Hypoxia

Ischemia is a condition that promotes a limitation of oxygen and glucose in the tissues due to the restriction of blood supply, mainly caused by blocked arteries [42]. It appears to be commonly associated with hypoxia. Fetal hypoxia refers to a generalized or localized oxygen insufficiency that may affect the fetuses in different ways during pregnancy [42], with most of the studies focusing on the consequences for the brain and heart.

Short periods of acute maternal uterine ischemia rapidly display severe cardiovascular alterations in fetuses with an unclamped umbilical cord. This is not observed in the case of fetuses with a clamped umbilical cord, which may indicate a reasonable resistance to hypoxia [43]. Nonetheless, we should not consider that acute maternal uterine ischemia is innocuous. There is also a large body of work in this area reporting that acute fetal hypoxia-ischemia without reoxygenation and repetitive hypoxia-reoxygenation lead to increased reactive nitrogen and oxygen species, which may be the basis of the fetal brain injury observed by the authors [42,44,45]. The decrease in the fetal heart rate (bradycardia) is also a common consequence of hypoxia [42,46]. Another possible outcome of fetal hypoxia is impaired motor control in newborn kittens because of subcortical motor pathway injury [47] or a reduction in gastrointestinal motility that may cause severe inflammation of the intestine (neonatal necrotizing enterocolitis) [48].

Based on evidence on how higher levels of free radicals and inferior antioxidant capacity play a role in the consequences of episodes of hypoxia-ischemia, some options to protect the fetuses were studied. Maternal antioxidant treatments (Supplementary Table S2) provided promising results, ameliorating fetal brain and myocardial injury [42,46].

### 2.5. Intrauterine Growth Restriction

Intrauterine growth restriction (IUGR) is responsible for about 20% to 50% of perinatal deaths, and, therefore, it is widely studied using rabbits as an animal model. It consists of a condition where fetuses fail to reach the expected rate of fetal growth [49]. Mild and severe IUGR can be induced with the ligation of unilateral uteroplacental vessels with a silk suture in a proportion of 20–30% and 40–50%, respectively [50]. IUGR can also be induced by restricting maternal food intake by 50% [51].

This condition can promote several problems, leading to a greater likelihood of stillbirths [52]. Prior research demonstrated that fetuses undergoing IUGR in the last period of gestation had lower placental and fetal weight, vascular congestion of the kidneys, and increased levels of reactive oxygen species (ROS) [53,54]. ROS can be a consequence of hypoxia, possibly induced by impaired renal vascularity [53]. Another study showed an increased expression of genes related to hypoxia and kidney development, function, and protection, such as the hypoxia-induced factor (HIF-1α), nuclear factor of activated T-cells 5 (NFAT5), interleukin 1 β (IL-1β), neutrophil gelatinase-associated lipocalin (NGAL), and ataxia telangiectasia mutated gene (ATM) [50].

Other studies have addressed the consequences of IUGR on the brains of rabbit fetuses, specifically focusing on the structural changes that have remained relatively unexplored until recent times, as well as on the cardiac function of these fetuses. As cited by Lopez-Tello and collaborators [8], the progression of cerebral development in rabbits, involving the maturation of motor skills, white matter, and myelination, bears similarity to that observed in humans. Simões et al. [55] described that rabbit fetuses undergoing severe IUGR had a smaller brain with metabolic alterations, more prevalent in the cortex than in the hippocampus and striatum. Also, IUGR may probably induce neuronal impairment based on the lower levels of aspartate and N-acetyl-aspartyl glutamate in cortex and hippocampus, and brain injury based on the higher levels of glycine in the striatum that were observed [55]. A previous study showed that the intensity of 18 metabolites in the tissue brain of severe IUGR fetuses differed significantly from the intensities observed in control fetuses. Evidence also suggests that those alterations may be dependent on the severity of the condition [54,56]. Regarding rabbit fetal brain structural alterations due to IUGR, Pla and collaborators [52] described that severe IUGR induces changes in the neuronal arborization pattern of the frontal cortex as well as abnormal oligodendrocyte maturation. Furthermore, a voxel-based analysis revealed fractional anisotropy differences in at least 10 different brain regions when comparing severe IUGR neonates and control neonates [57]. Severe IUGR may also promote a differential effect in ventricles since design-based stereology performed in IUGR rabbit fetuses revealed that only the left ventricle presented a significantly higher cardiomyocyte mean volume and a diminished number and length of cardiac capillaries [58]. If living, those rabbits are probably more vulnerable to cardiovascular diseases too [58].

Recently, a promising therapy was reported that aimed to overcome placental insufficiency [49]. This therapy is based on the intra-amniotic injection of a modified parental nutrient solution of glucose, amino acids, and electrolytes. When applied on the same day that severe IUGR was induced, even though this supplementation did not improve the fetal heart rate or birth weight of the IUGR fetuses, it increased their survival by 43% [49]. Moreover, using a rabbit model, undernourished from gestational day 9 onward, Lopez-Tello and collaborators [51] demonstrated that the vasodilator sildenafil citrate yielded promising outcomes as a therapeutic approach for IUGR. It improved placental development and led to fetuses with higher resistance and pulsatility indices in the middle cerebral artery, larger biparietal and thoracic diameters, and longer crown-rump lengths. However, there were also indications of possible blood overflow in the brain when using this molecule [51]. In humans, administering 50 mg of sildenafil citrate to mothers with pregnancies complicated by fetal growth restriction showed improvements in utero-placental blood flow [59]. This resulted in a significant prolongation of pregnancy, increased gestational age at delivery, improved neonatal weight, and reduced admissions to the neonatal intensive care unit [59].

Further biochemical-based studies are needed to determine IUGR biomarkers and which ones would be best suited for detecting the condition and monitoring the response to therapy [55]. Lopez-Tello et al. [8,60] have previously examined models concerning intrauterine growth restriction and fetal programming in rabbits. They emphasized rabbits as an excellent model organism for investigating pregnancy physiology [8]. Specifically, based on their findings, Lopez-Tello and colleagues suggest that using rabbits and underfeeding could be a useful approach to studying nutrient-related IUGR [60].

### 2.6. Superfetation

Superfetation is a rare reproductive disorder in which a new gestation occurs during an ongoing pregnancy, a condition already reported in numerous species, namely humans, rodents, fish, and livestock. Notwithstanding, no clear criteria exist for the identification of this condition. Other situations can still be mistaken for superfetation, including superfecundation, delayed nidation, variable pregnancy length, embryonic death and reabsorption, development retardation, and split parturition (reviewed in [61]).

The most recent possible case of superfetation in rabbits was reported in an obese doe accidentally remated some days after a confirmed pregnancy, in Mexico [21]. Since the fetuses were not responding to the manipulation for extraction, a cesarean was performed. Seven dead fetuses were observed, possibly due to secondary uterine inertia and dystocia, since one fetus was blocking the birth canal. No signals of infection were found. What caught the attention was the fact that, among the fetuses, six were overdeveloped, which may have contributed to the problems during labor, and one was severely underdeveloped and covered by a thick green fluid, called meconium, which delimits the intestines of the fetuses and is usually released only at the time of birth. Based on the morphological differences and mating records, the authors postulated that the underdeveloped fetus may have resulted from a superfetation, despite the unclearness regarding how a second implantation occurred [21]. Other studies have been reported previously: one described the case of a pregnant doe that mated a week before giving birth and a second healthy litter was born 23 days after the first [62], and another one described a case in which a doe gave birth twice within two weeks ([63] as cited by [61]).

The possibility that this condition may exist in some mammals as a reproductive strategy, namely in the European brown hare, closely related to the European rabbit, has already been considered. This hypothesis still raises numerous questions from an endocrine, evolutionary, or even immunological point of view, needing further investigation (reviewed in [61]).

## 3. Other Factors That Can Influence Fetal Development and Offspring Health and Behavior

Other factors, apart from pathologies, can play an important role in fetal programming, affecting the development and even kittens’ health in adulthood, and thus, should be considered. Some of those factors, such as the age, stress, and body condition of the doe, genotype, and intrauterine location of the fetuses, are briefly reviewed below.

### 3.1. Maternal Age

Advanced age is a factor widely reported as being related to a decline in fertility. Not only can it affect the capacity to maintain a pregnancy but the embryo itself is less likely to develop into a newborn [64].

A recent study, while showing that the does’ age highly influences fertility, demonstrated that this factor may be closely related to the number of parturitions too [65]. Nulliparous does showed a pregnancy rate of 69.8%, which increased continuously until reaching 4th parity with a pregnancy rate of 80%. From there on, the pregnancy rate started to decrease. Nonetheless, even older does can have healthy kittens. The same authors described the case of a farmed doe with 6 years that gave birth to 11 live-born kits [65]. However, according to our experience, the fertility rate of nulliparous does is above 90% if the breeding program is good and the does inseminated are sexually mature (more than 17 weeks of age) and have an adequate weight. A good rearing program for the young does include ad libitum feeding until the 12th week of age. Above this and until day 6 before AI (in general, during the 19th week of age), rabbit does must grow as established on the growth curve provided by the genetic companies, with an expected growth of 26 g/doe/day as a maximum. This is possible to achieve with a balanced feed, with a proper formulation, provided in a specific quantity per day to the animals. During this period, it is necessary to weigh the young does to evaluate their weight gain, and with this information, it is possible to adjust the amount of feed given per day. In addition, it is also important to apply a correct light program (intensity and number of h/day), avoid stress factors, and administer a good prophylactic program, which includes proper vaccination and deworming.

Protein modification can also be one of the causes of reduced fertility associated with age. Dicarbonyl species, for example, are well known for their capacity to promote posttranslational modifications of proteins, among others [66]. Recently, it was reported that the expression of a key enzyme for detoxification of reactive dicarbonyls, GLO1, was significantly reduced in the endometrium of older pregnant does (more than 108 weeks old) at the time of implantation, when compared with young does (16–20 weeks old) [67]. This probably implies that the uteri of older rabbits do not have a reasonable metabolic stress defense, which affects the pregnancy rate [67].

### 3.2. Maternal Stress

It is described that the hypothalamic-pituitary-gonadal (HPG) and the hypothalamic-pituitary-adrenal (HPA) axes are interrelated and influence each other’s functions when activated [68]. The HPG axis plays an important role in the regulation of reproductive functions, while the HPA axis, which is actively modulated by the gonadal steroids produced through the HPG axis, is essential for adapting to stress by adjusting the balance of hormones [68,69]. Both axes are affected by stress [68]. In this case, the HPA axis is activated, leading to the secretion of glucocorticoids and the mobilization of energy reserves [69]. However, an inappropriate or prolonged activation of the HPA axis can give rise to several disease states by inhibiting the secretion of gonadal hormones, such as testosterone and estrogen, which are important for reproductive functions [69]. Other mechanisms are also activated by stress, namely the release of prolactin and gonadal steroid hormones (reviewed in [69]).

The negative Impacts of maternal stress during pregnancy on fetus development have already been reviewed [70,71]. Nevertheless, further studies are required to comprehend the underlying mechanisms and the extent to which the consequences may reach [71]. Stress in pregnant does can trigger several outcomes, depending on the pregnancy period in which they are subjected to the stressful stimuli [72].

At least when exposed to noise stress, the consequences appear to be more severe if stress occurs until day 22 of gestation. When pregnant does were subjected to 30 min of electrical stimulus paired with the sound of a car horn on two consecutive days, either between the 4th and 16th days or the 16th and 22nd days of gestation, fetal death occurred. However, the same stimulus applied between the 22nd and 23rd days of gestation only caused growth delay, both the fetus and the organs, and, when applied from day 24 to day 25, it disturbed the weight of the organs. On the other hand, when the stress was induced at the end of the fetal period (from days 25 to 27), it increased the size of the placenta, accelerated the growth of the organs, and increased the fetuses’ weight when compared to controls. This interesting event can be an adaptative reaction of the fetus in response to stress [72]. Rabbits have very sensitive hearing and were already associated with a threshold area lying between 0 and 20 dB in the sensitive range. Few studies focused on the evaluation of the noise effect on rabbits’ reproduction, but rabbits killing their young, nervous abnormalities, behavior alterations, and traumatic injuries were already described (reviewed in [73]). In line with this, some authors recommend to house rabbits away from noise-generating operations [14]. On the other hand, some types of sound may have positive consequences for rabbits’ stress and, therefore, possibly on rabbits’ reproduction. A study in which rabbit colonies were enriched for 6 months with commercially available music CDs showed that the levels of fecal cortisol decreased during this period and increased again after the removal of the music. This indicates that rabbits were less stressed with music enrichment [74]. We believe that the consequences of noise on rabbits’ health and welfare will depend on factors such as decibels, frequency, and exposure time. Therefore, it is also possible that noise affects reproduction, both positively and negatively, as it seems to be related to stress levels in rabbits, but more studies are needed to define threshold areas regarding noise and its consequences on reproduction.

The handling of rabbits can also impact their well-being and, consequently, fetal development. Rabbits are delicate animals that can experience significant stress when subjected to human disturbance, known to induce physiological, immunological, hormonal, and behavioral changes that can affect reproduction, from ovulation to the safety and survival of the kits [17,75,76,77]. In another study, does that were 5–29 days pregnant were subjected to vibration stress and sound designed to simulate transport conditions. However, no detrimental effects were observed on gestation or the well-being of the newborns when compared to the control group. The only noticeable alteration was an elevated respiration rate in the does, which returned to normal levels within 20 min to 4 h [78].

On the other hand, the exposure of pregnant does to environmental stress, such as radiofrequency radiation (also emitted by wireless communication devices), may adversely affect offspring. A short-term exposure of 15 min per day during fetal tissue and organ maturation (between days 15 and 22 of pregnancy) resulted in differences in hepatic glucose regulation and the capacity of glutathione-dependent enzymes [79]. Glutathione serves as the primary endogenous antioxidant in most cells [80]. The findings also suggest that this radiation could induce cellular disruptions related to ROS and potential deficiencies in the intracellular antioxidant system [79].

Another environmental stressor is heat. While rabbits are known to adapt to a wide range of climates, their reproductive performance can still be influenced by thermal stress (reviewed in [16,18]). In a study conducted during a hot summer, reproductive parameters such as litter size and survival were more favorable for rabbits housed in underground shelters compared to those in conventional cages [81]. Additionally, research has shown that high temperatures could significantly reduce the number of gestations per year, from 10 liters to 4–5 ([82] as cited by [83]), and that maintaining rabbits at 30 °C was sufficient to induce prolonged or permanent impaired male fertility [84]. Therefore, temperatures exceeding 25 °C are not recommended, as they can have adverse effects on rabbits’ health and well-being since they will require the activation of homeostasis mechanisms. Moreover, rabbits may lose their ability to regulate their body temperature beyond 35 °C or a temperature-humidity index equal to or superior to 30 (reviewed in [16,83]). Therefore, even when housing rabbits outdoors, it is important to ensure adequate ventilation, shading, and access to fresh drinking water, namely when temperatures exceed 30 °C [85]. Air-conditioning systems can also be used to maintain a stable ambient temperature, particularly during the winter and summer, when temperatures could become more extreme [86].

Furthermore, Benedek and colleagues recently conducted a study on stress reactivity near birth that revealed a relationship between progesterone levels (an important hormone for nest construction) and cortisol levels. In comparison to does displaying consistently normal cortisol levels throughout parturition, more sensitive does that had elevated cortisol levels exhibited a noticeable delay in constructing their nests, despite maintaining the same nest quality. Moreover, these does gave birth to smaller litters, with an increased mortality rate registered. The authors believe that this negative impact can potentially be linked to disruptions in maternal behavior stemming from subtle hormonal regulatory changes [75].

### 3.3. Maternal Nutritional Status

**Figure 1:**
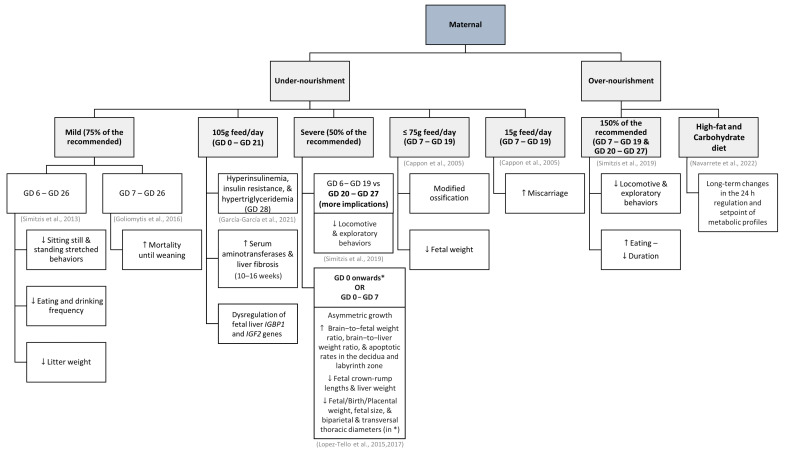
Examples of how maternal nutrition during gestation can affect offspring in rabbits [22,60,88,89,90,91,92,93,94]. ↑ increase; ↓ decrease.

The amount of food ingested by pregnant does was already mentioned regarding diabetes effects and prevention, but it has also been shown to influence the behavior of the kittens. In these cases, the outcome of the offspring depends on the magnitude of the variation in food intake and the gestation period in which these occur (summarized in Figure 1). It has also been demonstrated that the nutritional status of does before conception can play a role in this [87].

Mild maternal undernutrition (75% of the recommended, between the 6th and 26th day of gestation) can translate into kittens displaying decreased rates of sitting still and standing stretched behaviors, and eating and drinking with less frequency, but drinking for a longer time than kittens born to does fed ad libitum [88]. In recent studies that compared the consequences of ad libitum feeding throughout pregnancy (control) or dietary restriction (105 g/day) between day 0 and day 21 of gestation followed by ad libitum feeding until term, no significant differences were observed regarding the body weight or phenotype of their offspring [89,90]. However, fetuses from restricted-fed females had an altered metabolism, with higher levels of insulin and serum triglycerides on the 28th day of gestation, and higher serum levels of alanine transaminase and liver fibrosis during the juvenile period. Differences regarding kittens’ growth and other serum metabolic parameters were no longer evident in this period. It is worth mentioning that the does of the study group had a significantly higher food intake during the last week of gestation than the control does, which may indicate that this allowed the compensation of the previous food restriction, while also minimizing the consequences on kittens’ metabolism and development [89]. In another study by the same author, there was even a higher recorded implantation rate and a greater number of fetuses in the food restriction group (105 g/day, between days 0 and 21) compared to the ad libitum control group [90]. Although the fetuses from undernourished does showed dysregulated expression of the fetal liver *IGBP1* and *IGF2* genes, it appears that, overall, pre-implantation events and fetal development remained unaffected by the feeding restriction [90]. In other studies, it is also described that litter weight can be lower, although the weaning period seems sufficient to compensate for it [88,91]. Mild undernutrition between the 7th and 26th days of gestation can also promote higher rates of mortality until weaning [91].

Furthermore, severe maternal undernutrition (50% of the recommended) compromises offspring’s activity, namely locomotive or exploratory behaviors, while increasing resting behavior. The effects seem more severe when the food restriction is applied between the 20th and 27th days of gestation [92]. Compared to 150 g of feed per day, an intake of only 75 g or less per day between the 7th and 19th days of gestation has already been associated with changes in offspring ossification and reduced fetal weight, and an intake as low as 15 g of feed per day was already related to a higher incidence of miscarriage [22]. Lopez-Tello and collaborators [60] also demonstrated the detrimental impact of a 50% maternal food restriction throughout the entire gestation period. They noted that in comparison to the offspring of does fed ad libitum, this diet resulted in a noteworthy decrease in birth weight and fetal size, namely occipito-nasal length, as well as reduced crown-rump length and smaller biparietal and transversal thoracic diameters [60]. Later, Lopez-Tello et al. [93] explored the consequences of 50% maternal food restriction throughout the entire gestation period, as well as during the preimplantation period only (between days 0 and 7). In both groups, asymmetric growth was observed, with reductions in fetal crown-rump lengths, as observed before, and liver weight [93]. Additionally, the brain-to-fetal weight ratio, brain-to-liver weight ratio, and apoptotic rates in the decidua and labyrinth zones were increased [93]. Offspring of mothers who were undernourished throughout the entire gestation period also exhibited a significant reduction in fetal weight and placental weight [93]. Interestingly, their study suggests that food restriction during gestation could trigger strategic adaptations to preserve the bodyweight and health of the does, even if it results in growth restrictions for their offspring [93].

The offspring of over-nourished does (150% of the recommended energy requirements) can also move and explore less than the offspring of properly fed does, as well as eat more meals, albeit for a short time [92]. In a more recent study, the offspring of induced over-nourished does, who consumed a high-fat and carbohydrate diet prior to and during gestation, displayed significant long-term alterations in their 24 h serum metabolite patterns, with distinct differences between males and females [94].

These consequences may be related to stress induced by changes in nutritional status, namely undernutrition. In rodents and non-human primates, prenatal stress modified the response of offspring’s hypothalamic–pituitary–adrenocortical axis to stress and the brain neurotransmitter systems, affecting social behavior and anxiety, among others [95]. On the other hand, male offspring may also possibly suffer from impaired fertility due to a reduction inthe concentration of serum-free testosterone and gonadal weight induced by maternal hyperlipidic and hypercholesterolaemic diets during gestation and the weaning period [96].

### 3.4. Maternal Physical Condition

The physical condition is influenced by more than one factor, including the nutritional status, and can affect does’ capacity to get pregnant and maintain pregnancy. Body condition is commonly scored (BCS) based on a scale ranging from 1 (emaciated) to 9 (obese) [65]. According to a recent study that evaluated thousands of does between 1994 and 2019, BCS is mainly negatively correlated with the percentage of gestations [65]. Does with a BCS of 8, 7, 6, 5, 4, 3, and 2 had a pregnancy rate of 73.7%, 82.6%, 82.9%, 79.3%, 73.1%, 61.7%, and 35.3%, respectively. Although level five is generally considered the ideal score, here, the authors suggest that level six can be the optimum BCS, even if it is associated with being overweight [65].

However, other factors can have serious repercussions on the physical condition of does, affecting fertility and stillbirths. An important one is the intensiveness of reproductive cycles. In the case of animal production farms, does can be inseminated as regularly as every 33–34 days (intensive rhythm), but more commonly every 42 or 56 days (semi-intensive and extensive rhythms) [97]. A study by Theau-Clément showed that increasing the period of pause between parturition and the next insemination from 4 days to 11 or 18 days also increased total body weight and productivity, along with a decrease in kitten mortality [98].

### 3.5. Maternal and Embryonic Genotype

Genotypes vary between the different lines of rabbits, and it has already been suggested that uterine secretions can be modified by the embryonic genotype. Therefore, some years ago, it was postulated that prenatal survival does not depend entirely on maternal factors [99].

Later, other authors who evaluated two different lines selected for high and low uterine capacity and their interactions stated that fetal survival depends mainly on maternal genotype and suggested that the embryo genotype only influences fetal survival if the embryo is transferred to a favorable maternal environment [100]. Further studies were conducted, and Vicente and collaborators [101], who studied two lines of rabbits, one selected by growth rate and the other by litter size at weaning, collected evidence that both maternal and embryonic genotype influence the implantation rate and placenta weight, and that embryonic genotype influences fetal survival and weight. One year later, another study evaluated two rabbit lines: a synthetic one selected by individual selection for daily gain from weaning to slaughter since 1990 (R) and a New Zealand White line selected based on an index for litter size at weaning since 1980 (A) [102]. After testing the fixed effects of embryonic and maternal genotypes (R or A) and their interactions (R/R, R/A, A/R, A/A, [embryo/mother]), they concluded that the influence of both maternal and embryonic genotypes on implantation and fetal growth appears to change during gestation [102]. Interestingly, although embryonic genotype had an influence on prenatal survival, they did not observe an effect of the inbred lines (R/R and A/A) on fetal–placental gene expression (VEGF, ERBB3, TGFB2, IGF1, ITGA1, and INFG) on days 14 and 24 [102].

These different observations between studies may be due to genetic differences, as suggested by Vicente [101]. Embryonic genotype may have affected fetal and placenta weights in this last study mainly because the embryos and recipient does were from genetically different lines [101], while in the study of Mocé et al. [100], they used lines from the same base population.

### 3.6. Intrauterine Location

Previous research has demonstrated that the intrauterine location influences the growth and metabolic status of rabbit fetuses, including placental echotextures. Compared to cranial end fetuses, caudal fetuses showed higher mean placental grayness values, fetometric values, and pulsatile and resistance indices of the uterine artery (possibly due to the presence of a more vascularized uterine horn nearby the cervix). Ultrasound can play an important role since some placental alterations not detectable by physical examination can be detected with this image tool [9].

## 4. Monitoring Techniques

It is known that some events occurring during embryonic and fetal growth may impact the clinical history throughout life [6]. Monitoring techniques are therefore the best allies to diagnose pregnancy andensure a complete follow-up of the overall health of pregnant does and kittens during gestation [103]. In rabbit farming, the most commonly used technique for confirming pregnancy is palpation, typically performed from day 12 to day 15 after insemination to be reliable [14,104]. This traditional method involves manually assessing the does’ abdomen to detect changes in uterine structures and the presence of developing embryos. Nevertheless, it can be stressful, hurt the animal(s) in the case of heavy palpation, and is not always accurate due to food or gas in the bowels that may mislead the diagnosis [104]. Other advanced reproductive monitoring technologies are available, allowing for an earlier pregnancy diagnosis in rabbits, such as ultrasonography and radiography [14,104]. Nevertheless, palpation remains popular due to its simplicity and cost-effectiveness, as it requires no equipment [104]. An effort to develop new monitoring methods has been made recently, suggesting the application of Vis-NIR spatially resolved spectroscopy to distinguish pregnant rabbits from non-pregnant rabbit does. However, an improvement in sensitivity is still needed [105].

Apart from palpation, producers do not necessarily conduct regular pregnancy monitoring. Instead, they often resort to monitoring or screening in response to specific situations, such as when the number of births does not align with the animals’ genetic curve or when they encounter a high number of abortions on the farm. In some cases, serological tests or histologic analyses of tissues are also performed on specific animals as a diagnostic measure when there are suspicions of infectious diseases within the group, which can also lead to reproductive problems (reviewed in [14,15]).

However, the literature already describes various techniques for monitoring fetal development and assessing the overall health of fetuses in certain domestic species. These techniques include ultrasonography and, less commonly, radiography, both of which have applications beyond predicting pregnancy. Ultrasonography involves the use of Doppler ultrasound to monitor blood flow in the umbilical cord and placenta, as well as fetal heart rate monitoring to detect signs of distress (reviewed in [12]). Radiography can also be employed to assess fetal bone development [106,107].

As a more cost-effective alternative to conventional electrocardiograms (ECG), some commercially available monitors have emerged. These monitors detect the R-peaks of the ECG during recording and store inter-beat intervals. They have been widely applied in veterinary and behavioral research to also measure heart rate variability as a means of assessing stress and welfare in animals, which can, as discussed earlier, interfere with fetal development (reviewed in [13]).

Blood tests to evaluate hormone and inflammation marker levels during gestation are also documented [11]. In a recent study, various parameters, including serum concentrations of anti-Mullerian hormone (AMH), C-reactive protein (CRP), progesterone (P4), and complete blood count (CBC), were evaluated as markers for monitoring canine pregnancy [11]. The study’s findings suggested that assessing AMH and CRP levels could aid in determining the gestational stage and monitoring the progress of pregnancy. Additionally, combining these markers with ultrasound examinations and CBC results could prove beneficial [11].

Although less commonly utilized, amniocentesis can be employed to diagnose genetic abnormalities and determine the sex of the animal early. It is also described that an analysis of the components of the amniotic fluid would allow a better understanding of fetal metabolism and the identification of pathological conditions during pregnancy [108].

Furthermore, with ongoing research, new options may emerge for monitoring the health status of animals. This could include the use of remote devices and machine learning technologies, including sensors to predict when the animal will give birth. Such advancements have been recently reviewed in the context of cattle [109]. Yet, more research on these technologies and approaches is of utmost importance to establish and validate monitoring techniques for the animal species in which they will be used.

While these techniques can be valuable for good herd management, the cost and resources involved in implementing them can be significant. Moreover, it might not be necessary to evaluate every pregnant doe. Instead, producers can use these tools selectively to make a differential diagnosis of a certain pathology accurately based on a representative part of the herd if they observe consistent signals of potential problems in multiple does. This approach would allow producers to allocate resources effectively and ensure the well-being of their pregnant does and their offspring, ultimately benefiting both the animals and the enterprise.

On the other hand, owners of pet rabbits tend to invest more in their animals’ health monitoring. In these cases, it is more common to closely monitor pregnancies, often with the assistance of ultrasound examinations performed by a veterinarian, coupled with complementary analyses that can help assess the overall health status of the animal. Since ultrasonography remains one of the most complete and secure imaging tools available for feto-maternal monitoring, it will be further reviewed.

### 4.1. Ultrasonography

This medical imaging tool is based on the transmission of sound waves into the body using a probe, also called an ultrasound transducer. From the speed of sound in the tissue and the time each echo takes to return, the intensity and distances to the organs/tissues are calculated and displayed, making it possible to trace a two-dimensional image [10].

When considering which frequency to use, we must keep in mind that higher frequencies are more easily absorbed, so they do not penetrate as well as lower frequencies. Therefore, higher frequencies are better suited for analyzing superficial tissues and lower frequencies for deeper structures. Conventional imaging systems use ultrasound transducers with frequencies ranging between 5 and 12 MHz [110]. High-resolution ultrasound uses high-frequency waves, which have proven to be reliable and sensitive for monitoring the growth, morphology, and well-being of rabbit fetuses [10]. One of the major advantages is that it is a low-cost, safe, non-invasive, and easily repeatable method [10,111]. In turn, micro-ultrasound can be used in frequency ranges up to 50 MHz, allowing an exquisitely detailed anatomical evaluation at early gestational ages, including of umbilical vessels [112,113]. For this matter, this method can be considered superior to magnetic resonance imaging or lower-frequency ultrasound probes [112]. While using this tool, Dekoninck et al. [112] showed that fetal number and location made it easier to determine if the uterine horn was exteriorized, a procedure not advised in routine gestational diagnosis.

It has been described the diagnosis of pregnancy as early as the 6th–7th day of gestation in rabbits by ultrasonography using a 5–12.5 MHz linear-array, transcutaneous curve-linear, or ultrasonic transducers, among others [104,114,115]. It is worth noticing that this early diagnosis is based on the presence of fluid-filled, darkened structures inside the uterus. Those structures may be embryonic vesicles, but the fluid may also be present due to other conditions [103,116]. Even on days 9 and 10, embryonic vesicles may be very similar to a ball of feces; hence, an experienced technician is required [117]. However, an ultrasound by day 16 would already allow the identification of individual embryos and confirm viability [116]. Nonetheless, ultrasonography has a significant advantage.It can be performed both in real-time and offline, allowing the evaluation of static and dynamic processes, and no ionizing radiation is needed [112]. It is also reported that repeated ultrasound evaluationsdo not promote stress-induced abortion, at least under controlled conditions [6]. The use of 3D and 4D ultrasound for feto-maternal monitoring is still not widely used in small animals (reviewed in [12]).

To implement pregnancy monitoring in rabbits using ultrasound, it is important to know which values reflect normal development and good health and which do not. A study that monitored fetal growth during the second and last third of the pregnancy of healthy New Zealand rabbits after controlled mating, using ultrasound (estimated mean values in Supplementary Table S3), states that the biparietal diameter can be used to estimate the gestational age and is more adequate than the crown-rump length or trunk diameter [103].

Biparietal diameter can also be used as a parameter for fetal growth retardation. The authors also add that the 20th day of pregnancy may be a threshold stage for umbilical artery indices, namely for the resistance index that decreases significantly from this day, while the arterial pulsatility index is maintained [103]. Chavatte-Palmer and collaborators [6] also performed a comprehensive evaluation of fetal growth and placental viability in New Zealand does by ultrasound scanning.

A summary of some ultrasound applications for feto-maternal monitoring in rabbits is provided in Table 1.

### 4.2. Precautions before and during the Examination

First, to perform the examination, the fur of the ventral/inguinal area of the doe is commonly shaved and covered with a large quantity of hypoallergenic gel to enhance the transmission of sound waves [105].

Moreover, both the type of probe and the frequency should be selected according to the animal, tissue/organ, and area to be evaluated. For rabbits, priority should be given to micro-convex probes. Microtransducers are a subtype of probes that can be used for neonatal purposes due to their reduced aperture. Compared to larger probes, they allow more imaging flexibility [118].

Whenever possible, animals should be analyzed in the position in which they feel most comfortable, avoiding mechanical restraint, since maternal stress can induce changes in fetal development. In addition, some authors argue that a calm and quiet environment and a trained operator, as well as not exceeding four evaluations per pregnancy, are crucial aspects to reduce the likelihood of animal handling being stressful for the pregnant doe [6]. The large number of fetuses that each doe may carry can make the exam difficult and time-consuming. Therefore, Coombs et al. [10] suggest that the number of fetuses and organs to be studied should be determined in advance. The area and amount of fur to be shaved should also be considered, as pregnant rabbits tend to use their fur to line nests for kittens [116].

The mechanical index (MI), used to predict the occurrence of a non-thermal event, and the thermal index (TI), used to predict the greatest rise in tissue temperature for a given exposure, should also be taken into consideration [119]. Thermal and cavity effects can be minimized by performing a 1 min break pause between measurements [9]. Performing a pregnancy scan of only 5 min, with a pause of a few seconds every 60 s, at an MI and TI of 0.1, may also be an efficient protocol to avoid thermally induced teratogenesis in small animals such as rabbits [116]. This is important since fetuses of pregnant rabbits exposed to ultrasound for 60 min, whether in the first, second, or last third of the gestation, had significantly decreased levels of parathyroid hormone, probably due to a heat-induced hormonal disruption [120]. The above-mentioned guidelines aim to avoid stressing pregnant does and kittens or any disturbance to fetal or placental development.

## 5. Conclusions

This brief review provided a compilation of factors to consider when assessing maternal and fetal health and fetal development in rabbits. As mentioned, several factors have the potential to impact pregnancy since many biological pathways are involved. Consequently, it is not always easy to fully understand the causes of some events or even compare the results of different studies. At the same time, this is one of the reasons why it is essential to perform further studies.

Ultrasonography enables the monitoring of fetal viability and well-being, including the identification of fetal distress, hypoxia, and growth retardation. Thus, it should be applied more frequently in reproduction studies coupled with biochemical and (cyto)genetic screening. Future investigations should address the standardization of available monitoring techniques and the establishment of reference ranges. The comparison of the results between different pathologies, while considering the breed/strain, age, the number of births, and feeding and housing conditions used, will allow a better understanding of the mechanisms involved in the fetal development of rabbits and which values may reflect a good or bad prognosis for each case. By testing simultaneously other conditions that can influence pregnancy and evaluating other parameters than those previously described, it will also be possible to establish new biomarkers and therapies. Ultimately, feto-maternal monitoring throughout pregnancy in rabbits will allow the distinction between healthy or fertile rabbits and unhealthy or infertile rabbits. In addition, it will help to determine the most tailored prevention/treatment choice whenever feasible and which housing conditions are more prone to promote rabbits’ well-being and proper development.

In sum, pregnancy monitoring proves to be a helpful tool to assess fetal growth in rabbits, and both the techniques and findings of such studies show great potential to be applied in the rabbit production industry.

## Figures and Tables

**Table 1:** Some applications of ultrasonography in feto-maternal monitoring in rabbits. N/A—not applicable.

Imaging Tool		Technical Information	Animal Preparation	Sedation	Parameters Evaluated	Authors
High- Resolution ultrasound		<1 mm, using a high-resolution 12–5 MHz linear transducer	Anterior abdominal region shaved; supine position	No	Biometric data (fetal number and location; crown-rump length; femur length; renal length and circumference; biparietal diameter; abdominal circumference) and morphology (head, neck, face, thorax, lung, diaphragm, cardiac, abdomen, spine, extremities, genitalia, and fetal environment)	[10]
Doppler	Spectral	N/A	Anterior abdominal region shaved; supine position	No	Umbilical arteries: resistive index	[10]
			Ventral area shaved; left paralumbar fossa region examined		Uterine and umbilical arteries: pulsatility index and resistance index	[9]
					With B-mode (8.0 MHz; Gain: 97%; Deepness: 6 cm): Biparietal diameter; trunk diameter; and echotexture of placentas	
		Vivid q ultrasound (1.4–2.5 MHz phased array probe); 70 Hz high pass filter; Angle of insonation: <30º	Midline laparotomy and exteriorization of both uterine horns	Yes	Ductus venosus pulsatility index; aortic isthmus flow; left and right ventricular sphericity indices calculated as base-to-apex length/basal ventricular diameter; left ejection fraction cardiac output normalized by body weight	[58]
					With M-mode: longitudinal axis motion and annular peak velocities at the septal mitral and lateral tricuspid annulus (apical 4-chamber view); septal wall thickness (transverse 4-chamber view)	
	Color	Linear 7.5 MHz probe	Abdomen clipped; placed on their backs and prevented from moving in a custom-made case	No	Vesicle, placental, fetal length, and head size; umbilicus, kidneys, aortic diameter, limbs, among others made occasionally	[6]
	Pulsed	N/A	N/A	N/A	Blood flow in the ductus venosus (pulsatility index), umbilical vein, and umbilical artery	[112]
Micro-ultrasound		Linear 20 or 30 MHz probe; 30 µm resolution	Exteriorization of the uterine horn with the fetus of interest after a midline laparotomy; performed directly on the uterus constantly irrigated with warmed saline	Yes	Abdominal, cardiac, and thoracic circumferences; humerus and femur length; umbilical vein and artery diameter; deepest vertical pocket of amniotic fluid; and placental thickness	[112]

## Data Availability

No new data were created or analyzed in this study. Data sharing is not applicable to this article.

## Supplementary Materials

The following supporting information can be downloaded at: https://www.mdpi.com/article/10.3390/vetsci10100622/s1, Table S1: Medical conditions and how they affect rabbit pregnancy and fetal health and development; Table S2: Maternal antioxidant treatments tested in pregnant rabbits with induced hypoxia-ischemia; and Table S3: Mean values for the biparietal diameter (mm), trunk diameter (mm), fetal heart rate (bpm), and arterial pulsatility and resistance indices assessed by ultrasound between the 9th and 30th day of gestation of ten healthy New Zealand does, by Turna and Erdoğan (2016) [103]. The does were fed with standard pellets and became pregnant after controlled mating. All values were estimated based on the figures presented in their study. n/a—not applicable.
