# Supplementary material for: Pregnancy Complications and Feto-Maternal Monitoring in Rabbits
Source: Vet Sci. 2023 Oct 17;10(10):622. doi: 10.3390/vetsci10100622 (PMC10610772; doi:10.3390/vetsci10100622)
Supplement: Supplementary file 1 [file vetsci-10-00622-s001.zip › vetsci-2633450-supplementary.pdf]

Supplementary Table S1. Medical conditions and how they affect rabbit pregnancy and fetal health and development.*

MATERNAL HYPERTENSION		
**Placenta**		**Fetuses** [10]
▪ Alteration of the differentiation and gene expression [24]:<br>↓ 11β-hydroxysteroid dehydrogenase type 2 mRNA [GD 14];<br>↓ Angiotensin receptors type 1 and 2 mRNA [GD 28].	▪ ↑ Proportion and volume of placental fetal capillaries [24]:<br>-Mild hypertension: early-mid-gestation;<br>-Moderate hypertension: late-gestation.<br><br>▪ Affects the transference of nutrients to the fetuses [25];<br><br>▪ In late gestation [25]:<br>-Mild hypertension: ↑ Placental efficiency;<br>-Moderate hypertension: ↓ Placental blood flow.	▪ ↑ Fetal heart rate;<br>▪ ↑ Kidney length (GD 26);<br>▪ ↑ Biparietal diameter.

GESTATIONAL DIABETES		
**Blastocysts** [33]	**Placenta** [34]	**Fetuses** [34]
▪ Retarded growth, probably due to an impaired expression of:<br>-*Wnt* signaling molecules;<br>-Mesoderm-specific transcription factor, Brachyury.	▪ Irreversible structural and molecular adaptations.	▪ Retarded growth;<br>▪ Dyslipidemia;<br>▪ Hyperglycemia.

MATERNAL STRESS			
**Stress reactivity near birth** [75]**:** ↑ Cortisol levels in sensitive does, ↓ litters, ↑ Mortality rate, and delay in constructing their nests.<br>Consequences of stress: vary with the stimulus and the gestation period (examples below).			
**Noise** [72–74]	**Handling/Transport** [78]	**Environment**	
▪ Consequences appear to be more severe if stress occurs until GD 22: fetal death occurred.	▪ GD 5-29 (simulation with vibration and sound): ↑ Respiration rate in the does (returned to normal levels within 20 min to 4 h).	▪ Radiofrequency radiation (GD 15-22, 15 min/day) [79]: Differences in hepatic glucose regulation and the capacity of glutathione-dependent enzymes.	▪ Heat ([82] as cited by [83]):<br>↓ Litter size;<br>↓ Survival;<br>↓ Gestations per year.

**ECTOPIC GESTATION**		
**Prevalence/Diagnosis**	**Fetuses** [39]	**Next gestation**
▪ ↑ Prevalence: does subjected to artificial insemination [39].<br>▪ Rarely discovered, but some possible signs are [37,38]:<br>-Palpable mass(es);<br>-Lithopedia;<br>-Impaired mammary gland development (more studies needed).	▪ The majority was term size and well developed: would be viable if a cesarean section had been performed at the right time.	▪ Natural and abdominal gestations may occur simultaneously [39];<br>▪ New fetuses and dead fetuses may coexist [20,38].
**MATERNAL UTERINE ISCHEMIA AND FETAL HYPOXIA**		
**Fetuses**		
▪ Severe cardiovascular alterations in fetuses with unclamped umbilical cord after short periods of acute maternal uterine ischemia [43];	▪ ↑ Heart rate [42,46];<br>▪ ↓ Gastrointestinal motility (may cause neonatal necrotizing enterocolitis) [48];	▪ Brain injury [42,44,45];<br>▪ Subcortical motor pathways injury [47].
**INTRAUTERINE GROWTH RESTRICTION**		
**Placenta** [53]	**Fetuses**	
▪ ↓ Placental weigh.	▪ ↑ Stillbirths [52];<br>▪ ↓ Fetal weigh [53];<br>▪ Vascular congestion of the kidneys [50]<br>▪ ↑ Expression of genes related to hypoxia and kidney development, function, and protection: HIF-1α, NFAT5, IL-1β, NGAL, and ATM [50];<br>▪ ↓ Brain size [55];<br>▪ Metabolic alterations in the brain [55];<br>▪ Differential effect in ventricles [58].	

* ↑ - increased; ↓ - decreased; GD - Gestational day

Supplementary Table S2. Maternal antioxidant treatments tested in pregnant rabbits with induced hypoxia-ischemia.

Study	Condition & Treatment	Outcome
[42]	Repetitive ischemia-reperfusion<br>Maternal administration of a bolus of Trolox (T): 100 mg/kg dissolved in 5 mL/Kg 0.9% saline + continuous infusion of 50 mg/kg/h Trolox 5 mg/mL; OR Ascorbic acid (AA): 1,500 mg/kg dissolved in 5 mL/Kg 0.9% saline + continuous infusion of 60 mg/kg/h ascorbic acid 300 mg/mL; OR Trolox + Ascorbic acid (T+AA): doses described above.	Common to (T), (A), and (T+AA): Reduction of cell death in the fetal cortex and hippocampus; and greater antioxidant levels in the fetal brain; and (T+AA): less brain edema and liquefaction; and less hippocampal ischemic nuclei.
[45]	Sustained hypoxia-ischemia<br>Intraperitoneally administration of 2mg of Trolox (50 mg/Kg/dose) combined with 40 mg of Ascorbic acid (1,000 mg/Kg/dose), pH 7.4.	Improvement of the cellular viability and brain edema.
[46]	Hypoxia<br>Administration of a bolus of 100 mg/kg Trolox and 1,600 mg/kg Ascorbic acid (both dissolved in 5 mL/Kg 0.9% saline) with continuous infusion of 50 mg/kg/h of Trolox 5 mg/mL and 60 mg/kg/h of Ascorbic acid 300 mg/mL.	Slightly lower bradycardia during hypoxia and less myocardial damage after fetal hypoxia

Supplemental Table S3. Mean values for the biparietal diameter (mm), trunk diameter (mm), fetal heart rate (bpm), and arterial pulsatility and resistance indices assessed by ultrasound between the 9th and 30th day of gestation of ten healthy New Zealand does, by Turna and Erdoğan [103]. The does were fed with standard pellets and became pregnant after controlled mating. All values were estimated based on the figures presented in their study. N/A – not applicable.

**Parameter**	**Gestational day**						
	**9**	**13**	**16**	**20**	**23**	**27**	**30**
Biparietal diameter (mm) [r = 0.981, *P*<0.001]	N/A	4.0	6.8	9.1	11.7	14.2	16.9
Trunk diameter (mm) [r = 0.972, *P*<0.001]	N/A	6.3	10.0	14.5	19.7	23.5	N/A
Fetal heart rate (bpm) [r = -0.562; *P*<0.001]	175	210	235	255	235	220 (*P*<0.05)	245
Arterial pulsatility index [r = -0.383; *P*<0.01]	1.37	1.36	1.39	1.37	1.20	1.21	1.17
Arterial resistance index [r = -0.730; *P*<0.001]	0.87	0.85	0.88	0.83 (*P*<0.01)	0.75	0.70	0.71
